# Seasonal and trend variation of methane concentration over two provinces of South Africa using Sentinel-5p data

**DOI:** 10.1007/s10661-024-12871-0

**Published:** 2024-07-08

**Authors:** Swelihle Sinothile Sibiya, Paidamwoyo Mhangara, Lerato Shikwambana

**Affiliations:** 1 School of Geography, Archaeology and Environmental Studies, University of the Witwatersrand, Johannesburg, 2000 South Africa; 2 Earth Observation Directorate, South African National Space Agency, Pretoria, 0001 South Africa

**Keywords:** CH_4_, Eastern Cape, Mpumalanga, Sources, GIS and remote sensing, Emission

## Abstract

South Africa faces the urgency to comprehensively understand and manage its methane (CH_4_) emissions. The primary aim of this study is to compare CH_4_ concentrations between Eastern Cape and Mpumalanga regions dominated by cattle farming and coal mining industries, respectively. CH_4_ concentration trends were analyzed for the period 2019 to 2023 using satellite data. Trend analysis revealed significant increasing trends in CH_4_ concentrations in both provinces, supported by Mann–Kendall tests that rejected the null hypothesis of no trend (Eastern Cape: *p*-value = 8.9018e^−08^ and Mpumalanga: *p*-value = 2.4650e^−10^). The Eastern Cape, a leading cattle farming province, exhibited cyclical patterns and increasing CH_4_ concentrations, while Mpumalanga, a major coal mining province, displayed similar increasing trends with sharper concentration points. The results show seasonal variations in CH_4_ concentrations in the Eastern Cape and Mpumalanga provinces. High CH_4_ concentrations are observed in the northwestern region during the December-January–February (DJF) season, while lower concentrations are observed in the March–April-May (MAM) and June-July–August (JJA) seasons in the Eastern Cape province. In the Mpumalanga province, there is a dominance of high CH_4_ concentrations in southwestern regions and moderately low concentrations in the northeastern regions, observed consistently across all seasons. The study also showed an increasing CH_4_ concentration trend from 2019 to 2023 for both provinces. The study highlights the urgent need to address CH_4_ emissions from both cattle farming and coal mining activities to mitigate environmental impacts and promote sustainable development. Utilizing geographic information system (GIS) and remote sensing technologies, policymakers and stakeholders can identify and address the sources of CH_4_ emissions more effectively, thereby contributing to environmental conservation and sustainable resource management.

**Supplementary Information:**

The online version contains supplementary material available at 10.1007/s10661-024-12871-0.

## Introduction

It is widely acknowledged in the environmental monitoring community that global warming is one of the most urgent and pressing problems we face in the twenty-first century. This is due to the ever-increasing concentration of greenhouse gases (GHG) in the atmosphere (Yue and Gao, 2018). Despite the extensive research conducted by scientists and researchers to find solutions to manage global warming, there is still much to uncover and understand (Centre for Science & Environment, 2022; Smith & McDougal, 2017; de Souza Filho et al., 2019). Global warming leads to climate change, which can decrease the health standards of the population and affect other aspects of life (Centre for Science and Environment, 2022; Smith & McDougal, 2017; Shikwambana et al., 2022). Several gases are responsible for the increase in global warming, including carbon dioxide (CO_2_), CH_4_, ozone (O_3_), water vapor, and nitrous acids, among others (Centre for Science & Environment, 2022; Gorski, 2021). CH_4_ is the focus of this study, and its impacts are worse than those of CO_2_ despite its shorter lifespan in the atmosphere, which is only 11–12 years, making it easy to stabilize (Cahill & Swanson, 2023; Cahill et al., 2023; Dion, 2017). According to Cahill et al. (2023), CH_4_ contributes to ground level O_3_ concentration, which worsens human respiratory diseases. Cutting down on CH_4_ emissions in the near term and reducing its concentration in the atmosphere can slow the pace of global warming (Cahill & Swanson, 2023).

After CO_2_, CH_4_ is the second most crucial trace gas known to play a significant role in the greenhouse effect, contributing approximately 20% of its direct radiation force and having an estimated global warming effect 23 times that of CO_2_ (Nisbet et al., 2019; Niu et al., 2019). The literature highlights a limited number of activities as significant sources of CH_4_ emissions, which can be categorized as either natural or anthropogenic (Niu et al., 2019). Natural sources make up to 40% of the global CH_4_ emissions, and they include wetlands, floods, and wildfires, while anthropogenic sources include livestock, landfills, oil and gas extraction, biomass burning, and coal mining (Cahill et al., 2023; Dion, 2017). Since the beginning of anthropogenic evolution, almost half of the CH_4_ emissions have increased (Oxford Institute for Energy Studies, 2022; Shikwambana et al., 2022).

There are many gaps and uncertainties in the literature regarding CH_4_ emissions, and many of them are related to the type of data used. Niu et al. (2019) acknowledged that due to having limited datasets, they were unable to be more certain about their findings, and the causes of the increase in the CH_4_ emissions were not fully understood during the time of their study.

While most people are aware of the GHG and their effects, not everyone knows the significance of CH_4_ as a contributing gas, which may explain why it is difficult to keep CH_4_ gas under control (Armstrong et al., 2018). Despite decades of research, little progress has been made in improving scientific knowledge regarding the identification of current CH_4_ sources, sinks, and chemical mechanisms in the atmosphere. Consequently, solutions to the global CH_4_ issue have remained largely unchanged.

In South Africa, the Eastern Cape and Mpumalanga provinces represent contrasting landscapes, with the former characterized by extensive cattle farming and the latter dominated by coal mining activities (Department: Statistics South Africa, 2020). These industries are known to be significant sources of CH_4_ emissions, yet comprehensive studies comparing CH_4_ concentrations between regions dominated by cattle farming and coal mining are scarce in the context of South Africa.

Therefore, this aims to investigate CH_4_ spatial distribution and long-term trends in the Eastern Cape and Mpumalanga provinces. Moreover, this study further aims to compare CH_4_ concentrations between these regions. Through detailed spatial and temporal analyses, the study seeks to detect trends in CH_4_ concentrations and provide insights for targeted mitigation strategies. By interpreting the dynamics of CH_4_ emissions in these key regions, this research aims to inform policy decisions and contribute to efforts aimed at mitigating climate change and promoting sustainable development.

The hypothesis guiding this study is that the Eastern Cape province, known for its extensive cattle farming, will exhibit higher CH_4_ concentrations compared to Mpumalanga, which is dominated by coal mining activities.

## Materials and methods

### Study area

**Fig. 1:**
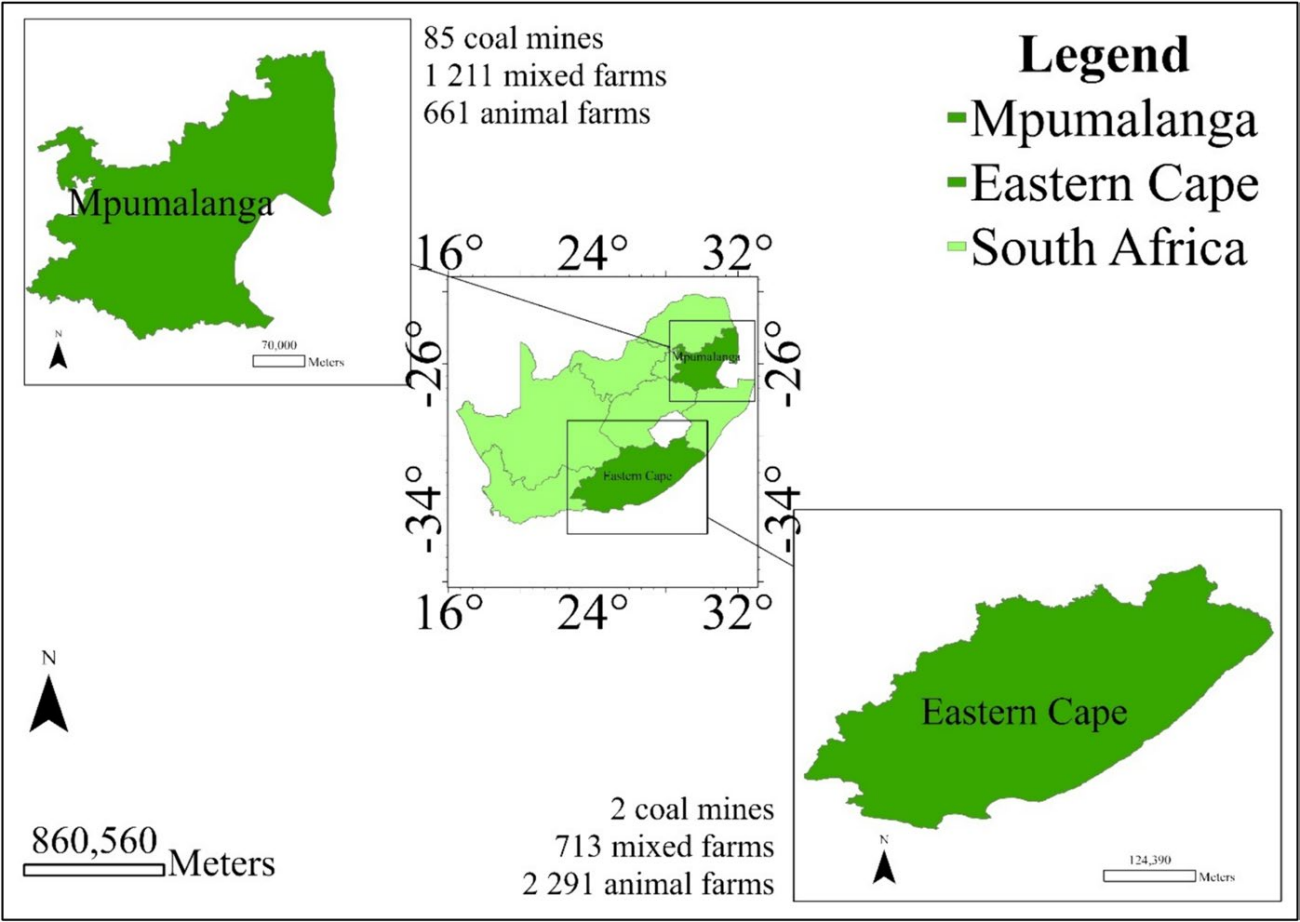
Map of South Africa showing the Eastern Cape and Mpumalanga provinces (data from Stats SA: https://www.statssa.gov.za/ (accessed: 11 March 2024); https://projectsiq.co.za/ (accessed: 11 March 2024))

The Eastern Cape (32.2968° S, 26.4194° E) and Mpumalanga (25.5653° S, 30.5279° E) provinces of South Africa offer compelling environments to study CH_4_ concentrations and the CH_4_ spatial distribution. Figure 1 shows a map of South Africa and the two provinces under study. The Eastern Cape’s diverse topography, climates, and agricultural practices, alongside its wetland ecosystems, provide a rich setting for understanding CH_4_ emissions (Zunckel & Perumal, 2013). In contrast, Mpumalanga, known for its prominent coal mining industry, warmer climate, and diverse land use presents a unique perspective on CH_4_ emissions (Mpe, 2019). These provinces provide valuable insights into both natural and anthropogenic CH_4_ sources.

### Data and method

#### Sentinel-5P (Precursor)

Google Earth Engine was used to gain access to Copernicus Sentinel 5 Precursor (S5P), the inaugural member of the Sentinel satellite family, which holds a special role in monitoring our atmosphere’s composition for applications like climate research, air quality assessment, and ozone tracking (Veefkind et al., 2020). At the heart of S5P is the TROPOMI (TROPOspheric Monitoring Instrument) payload, a highly advanced spectrometer designed in collaboration between The Netherlands and the European Space Agency (ESA) (Veefkind et al., 2020). TROPOMI operates across various parts of the light spectrum, including ultraviolet, visible, near infrared, and shortwave infrared (Veefkind et al., 2020). This exceptional instrument boasts a broad observation range spanning 2600 km, which allows it to capture data from the entire planet every day (Veefkind et al., 2020). It also maintains a remarkable level of detail, with spatial resolutions of approximately 3.5 × 5.5 km^2^, empowering it to effectively monitor our atmospheric conditions (Veefkind et al., 2020). More details on Sentinel-5P are found in Theys et al. (2019), Tilstra et al. (2020), and Verhoelst et al. (2021).

#### Mann–Kendall

The Mann–Kendall (MK) test is a non-parametric statistical test used to detect trends in time series data and ordered datasets (Conduent Healthy Communities Institute, 2022). It assesses whether a dataset displays a statistically significant trend, whether it is an upward or downward trend over time (Conduent Healthy Communities Institute, 2022). However, the Mann–Kendall test does not evaluate the extent of the observed change (Conduent Healthy Communities Institute, 2022). The result of the test is returned in *H* = 1 indicating a rejection of the null hypothesis at the alpha significance level (Fatichi, 2024). *H* = 0 indicates a failure to reject the null hypothesis at the alpha significance level (Fatichi, 2024). A small *p*-value (e.g., < 0.05) suggests statistical significance, indicating the presence of a significant trend (Conduent Healthy Communities Institute, 2022). Appendix 1 gives a full definition of the Mann–Kendall test.

## Results and discussion

### CH_4_ spatial distribution and concentration

**Fig. 2:**
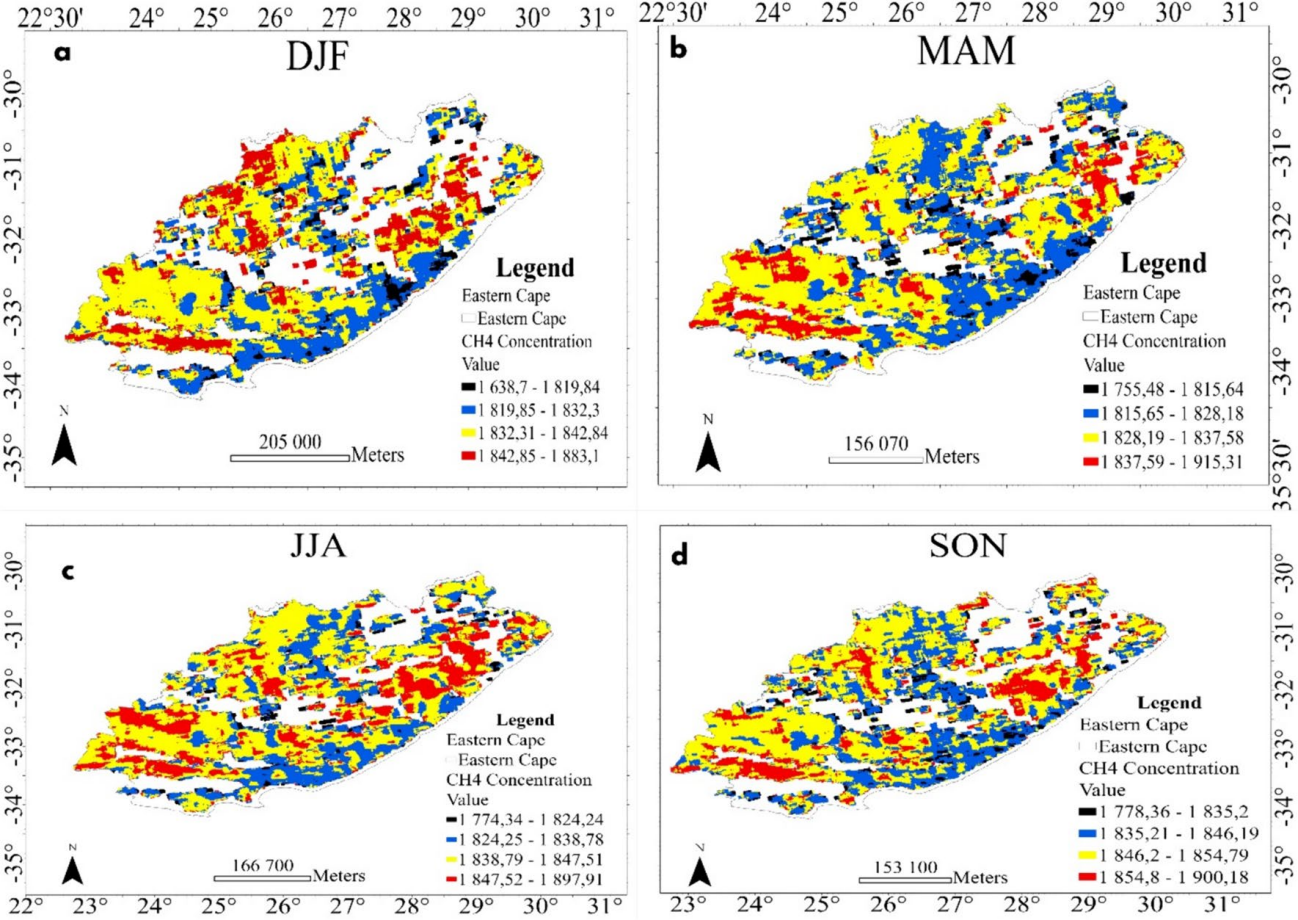
Spatial distribution of CH_4_ concentrations in the Eastern Cape province extracted from Google Earth Engine for the period 2019–2023

**Fig. 3:**
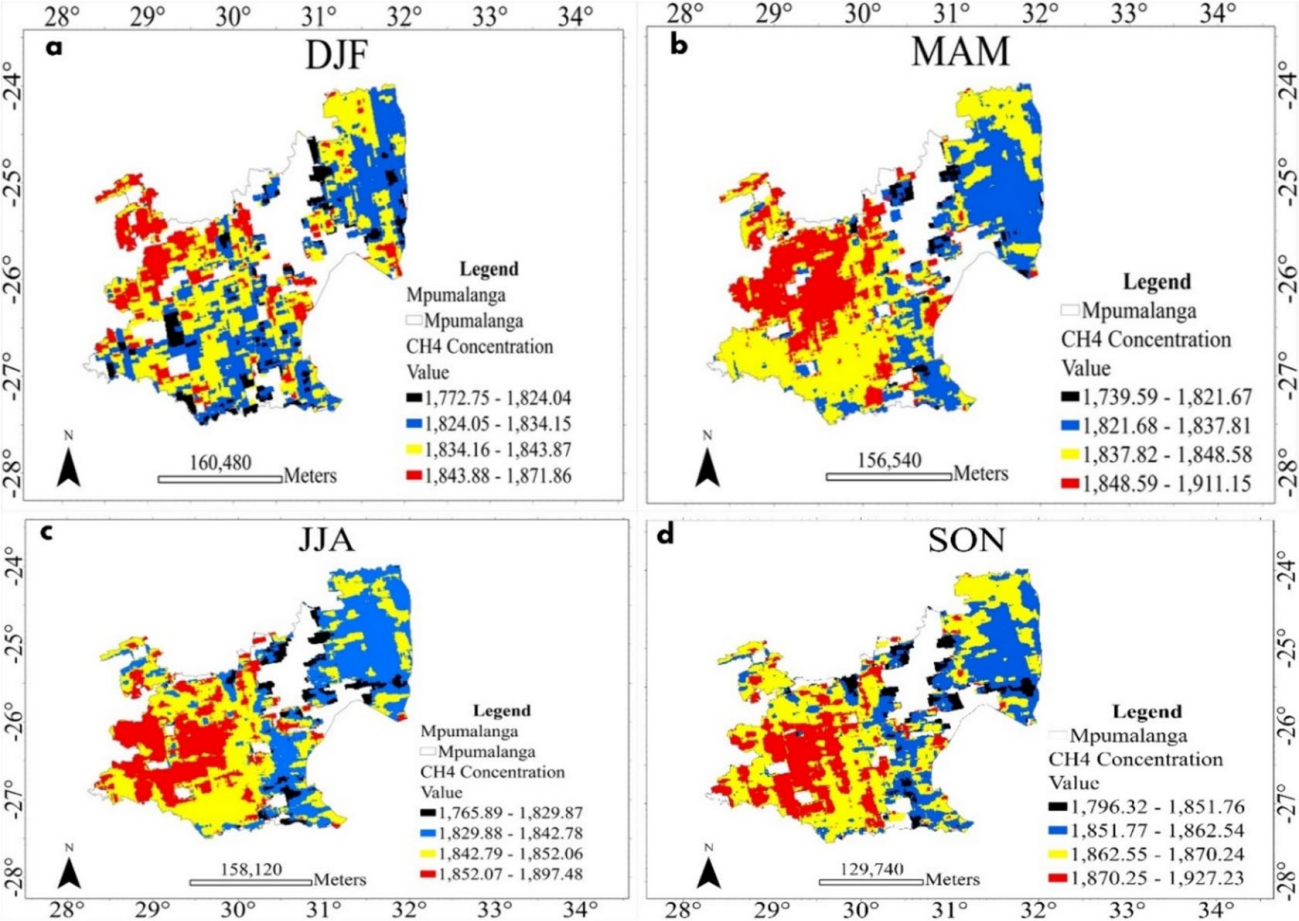
Spatial distribution of CH_4_ concentrations in the Mpumalanga province extracted from Google Earth Engine for the period 2019–2023

The seasonal spatial distribution of CH_4_ concentration over the Eastern Cape and Mpumalanga provinces is shown in Figs. 2 and 3, respectively. The analysis was conducted for each of the four weather seasons: summer (DJF), autumn (MAM), winter (JJA), and spring (SON). There are observed variations in CH_4_ concentrations across different seasons in the Eastern Cape province that provide insights into the spatial and temporal dynamics of CH_4_ emissions (see Fig. 2a–d). The dominant blue line along the coastal border indicates stable, moderately low concentrations influenced by oceanic factors and coastal vegetation, while black patches suggest areas of extremely low concentrations, possibly due to sparse vegetation cover or efficient CH_4_ oxidation processes (Hamdan & Wickland, 2016; Science Daily, 2022). Additionally, extensive water bodies in coastal regions act as sinks, absorbing and dissolving CH_4_, while coastal vegetation further mitigates emissions by consuming CH_4_ (Hamdan & Wickland, 2016; Science Daily, 2022). Significant changes in CH_4_ concentrations in the southeastern region, with peaks in DJF (see Fig. 2a), highlight the influence of weather and climate. Similarly, high concentrations in the northwestern region during DJF, followed by shifts to lower concentrations in MAM (see Fig. 2b) and JJA (see Fig. 2c), highlight the complexity of seasonal influences and anthropogenic activities since winter can be linked to a decrease in human activity with everyone spending most of their time indoors (Etiope & Sherwood Lollar, 2013; Zheng et al., 2019; May, 2020). The presence of a faint blue line across the province suggests spatial distribution influenced by land use practices and soil characteristics.

Meanwhile, in Mpumalanga, there is a dominance of high CH_4_ concentrations in southwestern regions and moderately low concentrations in the northeastern regions, observed consistently across all seasons of the year (see Fig. 3a–d). The southwestern regions of Mpumalanga fall within the Highveld, characterized by higher elevations and cooler temperatures compared to the northeastern Lowveld (Siyabona Africa, n.d.a). Cooler temperatures in the Highveld may contribute to slower rates of CH_4_ oxidation, leading to the accumulation of CH_4_ in the atmosphere and higher concentrations observed in satellite imagery across all four counts as observed in Fig. 3a–d (Hamdan & Wickland, 2016; Science Daily, 2022). Additionally, the southwestern Highveld regions are often associated with extensive agricultural activities, including crop cultivation and livestock farming, which are significant sources of CH_4_ emissions (Siyabona Africa, n.d.b). Seasonal factors, such as temperature inversions during MAM (Fig. 3b) and JJA (Fig. 3c), can trap pollutants closer to the surface, leading to higher observed concentrations in satellite imagery (Wei et al., 2023). Saunois et al. (2016) also showed that CH_4_ seasonal variation depends on the production resources such as wetlands, rice fields, and burning biomass and the outflow from the atmospheric reaction of this gas. Furthermore, a difference in the seasonal distribution can be caused by a combination of the following drives: (a) higher temperatures cause higher methane emissions, (b) higher vegetation cover causes lower methane emissions, and (c) high moisture levels cause lower methane emissions (Javadinejad et al., 2019). Differences in vegetation types and soil conditions between the Highveld and Lowveld regions also play a role in CH_4_ emission differences observed in the satellite image Fig. 3a–d (Hamdan & Wickland, 2016; Science Daily, 2022).

### Trend analysis

**Fig. 4:**
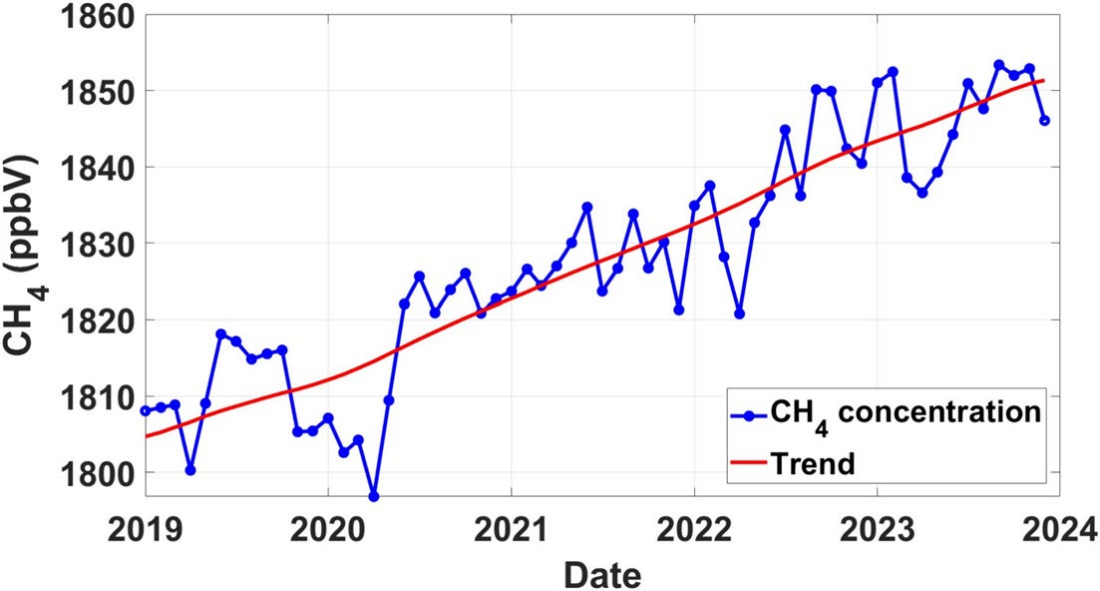
Temporal trend analysis of CH_4_ concentrations in the Eastern Cape province derived from MATLAB using Sentinel-5P data from 2019 to 2023

**Fig. 5:**
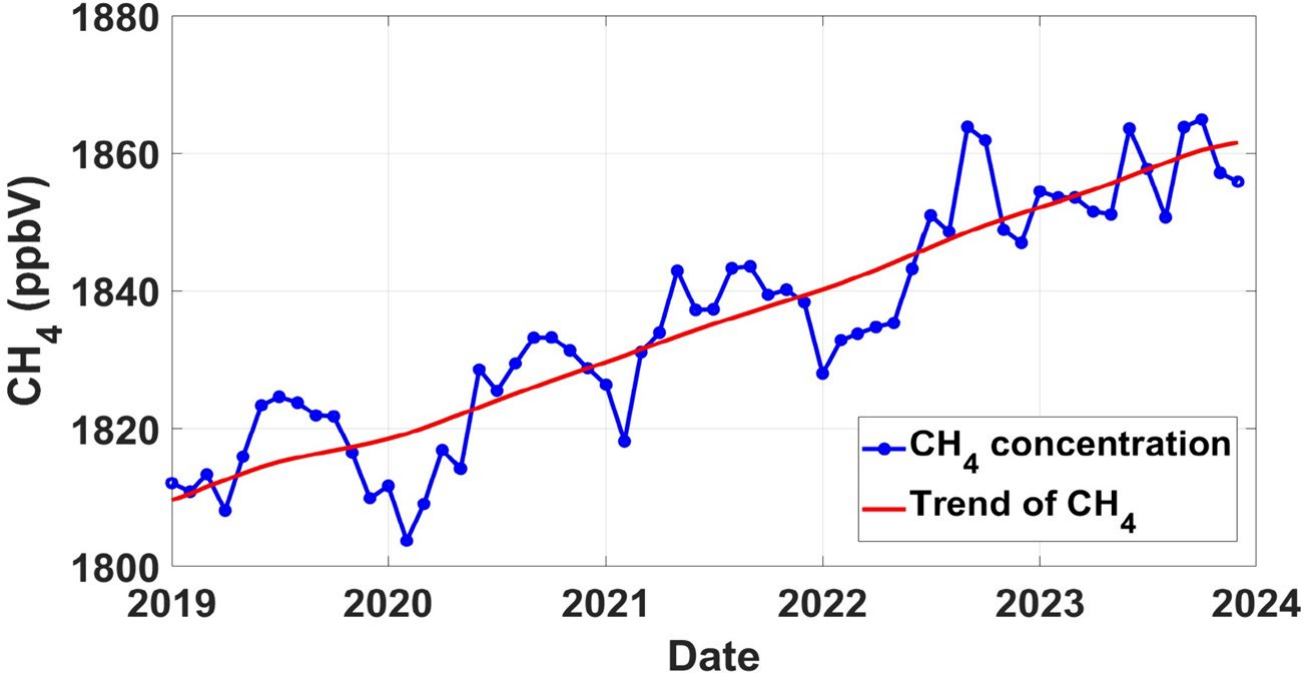
Temporal trend analysis of CH_4_ concentrations in the Mpumalanga province derived from MATLAB using Sentinel-5P data from 2019 to 2023

The trend analysis results for CH_4_ concentrations in the Eastern Cape and Mpumalanga provinces (see Figs. 4 and 5) reveal intriguing patterns with significant implications for understanding CH_4_ dynamics in these regions. The trend line for Eastern Cape (see Fig. 4) displays an increasing trend over time, with sharper points rather than smooth curves when concentrations decrease after reaching their peaks. Sharp trend lines suggest abrupt fluctuations in concentration levels, which could be influenced by various factors such as seasonal variations, changes in emission sources, or external environmental factors (Wei et al., 2023). The concentration reached a peak of 1852 ppbV in February 2023, which can be attributed to a variety of factors such as changes in land use and other factors that contribute to elevated emissions (Tate, 2015). Furthermore, the concentration distribution in Eastern Cape appears to fluctuate within a narrower range compared to Mpumalanga, with concentrations remaining relatively stable between peaks. This may be as a result of the dominant influence of specific emission sources and environmental conditions unique to the province. This is a call for further research on this.

In the Mpumalanga province, the trend line exhibits a consistent increase over the analyzed period, with concentrations reaching a peak of 1864 ppbV in September 2022 (see Fig. 5). The increase in the trend is likely due to the slow removal of CH_4_ in the atmosphere; thus, the concentration builds up with time. Notably, each year experiences a cyclical pattern, with concentrations peaking and subsequently decreasing, forming noticeable curves in the data (see Fig. 5). Each year, there is a distinct cycle where concentrations gradually increase to reach a peak before subsequently decreasing. These fluctuations form noticeable curves in the data, indicating a periodic rise and fall in CH_4_ levels. This pattern implies that CH_4_ emissions in the Eastern Cape exhibit seasonal variability, with concentrations rising and falling over the given period.

The differences in CH_4_ concentration trends between the Eastern Cape and Mpumalanga provinces may likely be a reflect variations in emission sources, environmental conditions, and land use practices between the two regions. Mpumalanga’s cyclical patterns and smoother concentration curves may suggest a more complex interplay of natural and anthropogenic factors influencing CH_4_ emissions (see Fig. 5).

Based on the results presented in Figs. 4 and 5, it appears that Mpumalanga has the highest CH_4_ concentration compared to the Eastern Cape. While both provinces exhibited increasing trends in CH_4_ concentrations over the study period which is in line with the global CH_4_ increase, the peaks observed in Mpumalanga (1864 ppbV) were higher than those in the Eastern Cape (1852 ppbV). Additionally, the sharper concentration points observed in Mpumalanga suggest a more intense emission source compared to the cyclical patterns observed in the Eastern Cape. Therefore, based on the trends and patterns identified, Mpumalanga can be assumed to have the highest CH_4_ concentration among the two provinces.

### Mann-Kendell

The Mann–Kendall test results reveal significant trends in CH_4_ concentrations for both the Eastern Cape and Mpumalanga provinces of South Africa (see Appendix 2). In both cases, the null hypothesis, which assumes no trend in the data, is rejected based on the calculated *p*-values, indicating the presence of a significant trend in CH_4_ concentrations over the analyzed period.

For the Eastern Cape, the *p*-value of 8.9018e^−08^ suggests a highly significant trend in CH_4_ concentrations, leading to the rejection of the null hypothesis. This result confirms the observed increasing trend in CH_4_ concentrations in the region, as indicated by the time series analysis (see Fig. 4). Similarly, in Mpumalanga, the Mann–Kendall test yields a *p*-value of 2.4650e^−10^, indicating a highly significant trend in CH_4_ concentrations (see Table 1). This result also aligns with the observed trend of increasing CH_4_ concentrations in Mpumalanga, as highlighted in the time series analysis (see Fig. 5). The rejection of the null hypothesis emphasizes the urgency of addressing CH_4_ emissions in both provinces, where concentrations are also on the rise and pose potential environmental and health risks.

**Table 1:** Mann-Kendall test for CH_4_ concentrations in Eastern Cape and Mpumalanga; (*p*-value < 0.05)

Parameter	Hypothesis (H)	*P*-value	Null hypothesis (H_0_)
Eastern Cape	1	8.9018e^-08^	Rejected, significant trend.
Mpumalanga	1	2.4650e^-10^	Rejected, significant trend.

In the context of the discussed CH_4_ trend analysis, these Mann–Kendall test results provide statistical confirmation of the observed trends in CH_4_ concentrations in both provinces. The significant trends identified highlight the need for proactive measures to mitigate CH_4_ emissions and address the underlying factors driving these trends. By recognizing and addressing the sources of CH_4_ emissions, policymakers and stakeholders can work towards containing the escalating concentrations of CH_4_ in both the Eastern Cape and Mpumalanga, thereby mitigating environmental impacts and promoting sustainable development.

## Conclusion

The comparison between the Eastern Cape and Mpumalanga highlighted unexpected patterns in CH_4_ concentration dynamics. Overall, the study shows notable seasonal variations in CH_4_ concentrations in the Eastern Cape provinces. High CH_4_ concentrations are observed in the northwestern region during the DJF season, while lower concentrations are observed in the MAM and JJA seasons in the Eastern Cape province. In the Mpumalanga province, there is a dominance of high CH_4_ concentrations in southwestern regions and moderately low concentrations in the northeastern regions, observed consistently across all seasons. The study also showed an increasing CH_4_ concentration trend from 2019 to 2023 for both provinces.

The results indicated comparable or even higher CH_4_ concentrations in this province compared to the Eastern Cape. Coal production releases methane trapped in coal seams and surrounding strata. Coal mine methane is closely linked with coal production; once production is halted and the mine is abandoned, it continues to release methane over a long period of time (Kholod et al., 2020). This is one of the reasons why there are higher concentrations of methane in Mpumalanga than in the Eastern Cape province. This unexpected finding challenges conventional assumptions about CH_4_ emission sources and highlights the complexity of environmental factors influencing emissions.

These findings have broader implications for understanding CH_4_ emissions and climate change mitigation efforts. By uncovering unexpected trends in CH_4_ concentrations, this study emphasizes the importance of continued research and monitoring to inform evidence-based policy decisions. Furthermore, it underlines the need for comprehensive data collection, including ground-based measurements, to enhance our understanding of CH_4_ emission sources and dynamics.

While unexpected, the findings of this study contribute to the broader discourse on CH_4_ emissions and highlight the importance of ongoing research to address climate change challenges effectively. By refining our understanding of CH_4_ emission sources and dynamics, we can develop more targeted mitigation strategies and work towards achieving sustainable environmental management practices.

### Supplementary Information

Below is the link to the electronic supplementary material.

Supplementary file1 (DOCX 34.3 KB)

## Data Availability

No datasets were generated or analyzed during the current study.
