# Supplementary material for: Seasonal and trend variation of methane concentration over two provinces of South Africa using Sentinel-5p data
Source: Environ Monit Assess. 2024 Jul 8;196(8):713. doi: 10.1007/s10661-024-12871-0 (PMC11230962; doi:10.1007/s10661-024-12871-0)
Supplement: Supplementary file 1 — Supplementary file1 (DOCX 34.3 KB) [file 10661_2024_12871_MOESM1_ESM.docx]

**Appendix 1**

The Mann-Kendall test is based on the statistic $S$ defined as follows:

$S=\sum_{i=1}^{n-1} \sum_{j=i+1}^{n} sign\left(X_{j}-X_{i}\right)$ (1)

Where $n$ is the number of data points, $x_{j}$ and $x_{i}$ are data values at time $j$ and $i\left(j>i\right)$, respectively. Denoting $x=\left(x_{j}-x_{i}\right)$

$sign(x)\left\{ \begin{aligned} +1, ifx>1 \\ 0, if x=0 \\ -1, if x<1 \end{aligned} \right.$ $\left\{ \begin{aligned} +1, ifx>1 \\ 0, if x=0 \\ -1, if x<1 \end{aligned} \right.$ (2)

For large samples $\left(n>10\right)$, the sampling distribution of $S$ is assumed to be normally distributed with zero mean and variance as follows:

$Var\left(S\right)=\frac{n\left(n-1\right)\left(2n+5\right)-\sum_{i}^{n} t_{i}\left(t_{i}-1\right)\left(2t_{i}+5\right)}{18}$ (3)

Where $n$ is the number of tied (zero difference between compared values) groups and $t_{i}$ is the number of data points in the $i$the tied group. The H-statistic or standard normal deviate is then computed by using equation:

$Z=\left\{ \begin{aligned} \frac{S-1}{\sqrt{Var\left( S \right)}} if S>0 \\ 0 if S=0 \\ \frac{S+1}{\sqrt{Var(S)}} if<0 \end{aligned} \right.$ $H=\left\{ \begin{aligned} \frac{S-1}{\sqrt{\mathrm{Var}\left( S \right)}} if S>0 \\ 0 if S=0 \\ \frac{S+1}{\sqrt{Var(S)}} if<0 \end{aligned} \right.$ (4)

If the computed value of $Z>Z_{\frac{\alpha}{2}}$, then the null hypothesis of no trend is rejected at $\alpha$ level of significance in a two-sided test (i.e. the trend is significant). In this study, the null hypothesis was tested at 5% significance level.

**Appendix 2**

**Table A1.** Mann-Kendall test for CH_4_ concentrations in Eastern Cape and Mpumalanga; (p-value <0.05)

| Parameter | Hypothesis (H) | P-value | Null hypothesis (H_0_) |
| --- | --- | --- | --- |
| Eastern Cape | 1 | 8.9018e-08 | Rejected, significant trend. |
| Mpumalanga | 1 | 2.4650e-10 | Rejected, significant trend. |
